# The Known and Potential Intersections of Rab-GTPases in Human Papillomavirus Infections

**DOI:** 10.3389/fcell.2019.00139

**Published:** 2019-08-14

**Authors:** Jesse M. Young, Amira Zine El Abidine, Ricardo A. Gómez-Martinez, Michelle A. Ozbun

**Affiliations:** ^1^Department of Molecular Genetics and Microbiology, University of New Mexico School of Medicine, UNM Comprehensive Cancer Center, Albuquerque, NM, United States; ^2^Department of Obstetrics & Gynecology, University of New Mexico School of Medicine, UNM Comprehensive Cancer Center, Albuquerque, NM, United States

**Keywords:** HPV, virus entry, Rab5, Rab7, virus infection, epithelial biology, Rab9A, Rab6A

## Abstract

Papillomaviruses (PVs) were the first viruses recognized to cause tumors and cancers in mammalian hosts by Shope, nearly a century ago (Shope and Hurst, 1933). Over 40 years ago, zur Hausen (1976) first proposed that human papillomaviruses (HPVs) played a role in cervical cancer; in 2008, he shared the Nobel Prize in Medicine for his abundant contributions demonstrating the etiology of HPVs in genital cancers. Despite effective vaccines and screening, HPV infection and morbidity remain a significant worldwide burden, with HPV infections and HPV-related cancers expected increase through 2040. Although HPVs have long-recognized roles in tumorigenesis and cancers, our understanding of the molecular mechanisms by which these viruses interact with cells and usurp cellular processes to initiate infections and produce progeny virions is limited. This is due to longstanding challenges in both obtaining well-characterized infectious virus stocks and modeling tissue-based infection and the replicative cycles *in vitro*. In the last 20 years, the development of methods to produce virus-like particles (VLPs) and pseudovirions (PsV) along with more physiologically relevant cell- and tissue-based models has facilitated progress in this area. However, many questions regarding HPV infection remain difficult to address experimentally and are, thus, unanswered. Although an obligatory cellular uptake receptor has yet to be identified for any PV species, Rab-GTPases contribute to HPV uptake and transport of viral genomes toward the nucleus. Here, we provide a general overview of the current HPV infection paradigm, the epithelial differentiation-dependent HPV replicative cycle, and review the specifics of how HPVs usurp Rab-related functions during infectious entry. We also suggest other potential interactions based on how HPVs alter cellular activities to complete their replicative-cycle in differentiating epithelium. Understanding how HPVs interface with Rab functions during their complex replicative cycle may provide insight for the development of therapeutic interventions, as current viral counter-measures are solely prophylactic and therapies for HPV-positive individuals remain archaic and limited.

## Introduction

### HPVs in Human Disease

Human papillomaviruses (HPVs) are small, non-enveloped icosahedral viruses of 55 nm containing a circular, ≈8-kb double-stranded DNA genome condensed by cellular histones. All PVs cause benign epithelial hyperproliferative diseases and tumors in mucosal or cutaneous epithelial sites as part of their normal replicative processes. As strictly human pathogens, HPVs have a narrow tropism for human keratinocytes and can only complete their replicative cycles in stratifying and differentiating squamous epithelium (Doorbar et al., 2012). Many HPV infections are inapparent, but certain HPV types cause symptomatic hyper-proliferative lesions (i.e., tumors, warts, or papillomas). Typically, HPV-induced lesions are self-limiting, and eventually cleared by a competent host immune system. However, some individuals fail to clear lesions, and if uncontrolled the lesions can cause clinical morbidity. Of the 225 recognized HPV genotypes, a handful termed “high-risk” or “oncogenic” are found associated with squamous cell or adeno-carcinomas (Van Doorslaer, 2013; Van Doorslaer et al., 2013; Bzhalava et al., 2014). Approximately 15 high-risk HPVs, including HPV types 16, 18, 31, 33, 45, 52, and 58, are etiologically linked to cervical, anogenital, and oropharyngeal cancers. HPVs with a low-risk of causing malignant carcinomas comprise the majority of the known HPV genotypes (Egawa and Doorbar, 2017). However, the world-wide morbidity caused by low-risk HPVs cannot be overstated. Low-risk HPV types 6 and 11 most generally cause benign anogenital or laryngeal warts. Although these lesions infrequently progress to malignancies, they often require multiple clinical interventions, with treatment strategies advancing little over the last century. Despite the availability of effective vaccines that prevent infections by mucosal HPV genotypes 6, 11, 16, 18, 31, 33, 45, 52, and 58, many people remain unvaccinated and therapeutic approaches have yet to be successful in curing persistent HPV infections. Overall, HPVs are the most common sexually transmitted infectious agents with ≥80% of sexually active, unvaccinated individuals acquiring one or more genital HPV infection in their lifetimes. Oncogenic HPVs are responsible for ≈35% of all pathogen-linked cancers (Elgui de Oliveira, 2007) and nearly 5% of the total worldwide cancer burden (Forman et al., 2012; Schiffman et al., 2016).

### Overview of the Replicative Cycle of HPVs

**FIGURE 1:**
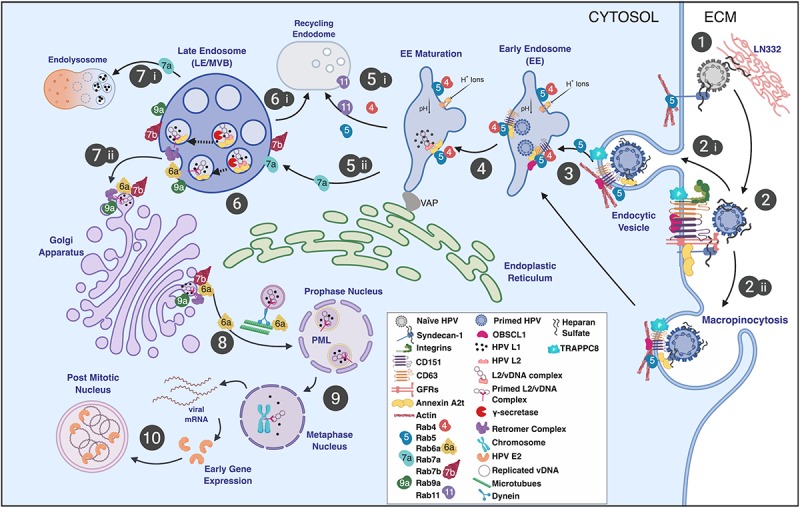
Schematic representation of the HPV infectious entry pathway in keratinocytes noting the involvement of Rab-GTPases in HPV trafficking. (step 1) HPV virions bind the extracellular matrix (ECM), basement membrane, and/or plasma membrane via HSPGs (e.g., syndecan-1) and laminin-332 (LN332). (step 2) Attached virions are conformationally altered by host enzymes, released from the plasma membrane or ECM, and translocate to tetraspanin-enriched microdomains containing a putative uptake receptor complex (e.g., CD151, CD63, integrins, A2t, EGFR, etc.). Viral uptake is thought to occur via (step 2i) receptor-mediated endocytosis, (step 2ii) through a process similar to macropinocytosis, defined as clathrin-, caveolin-, dynamin-, cholesterol-, flotillin-, and lipid raft-independent. (step 3) Entry is facilitated by actin polymerization and remodeling with the involvement of CD151, CD63, adaptor proteins (e.g., OBSCL1 and syntenin-1), A2t, and TRAPPC8 leading to virion localization to EE. (step 4) Viral localization to EE is thought to be CD63 and Rab5 dependent and is coupled to the acidification of the EE. (step 5) Endosomal tubulation ensues with the formation of an ER contact via the VAP complex. EE acidification results in capsid dissociation, releasing the viral genome in a complex with L2. EE have multiple fates and can either mature into recycling endosomes (step 5i) or LE/MVB (step 5ii). Endosomal maturation is linked to Rab function and is defined by Rab conversion (step 5i,5ii; see the text for details). (step 6) Intraluminal L2 is cleaved by γ-secretase exposing the L2 cell-penetrating peptide (CPP) and a transmembrane domain. MVB sorting mechanisms give rise to recycling endosomes, which are not known to be involved in HPV intracellular trafficking (step 6i). (step 7i) L2 membrane penetration and exposure of the cytosolic domain of L2 leads to the recruitment of the retromer complex. Retromer and Rabs 6a, 7b, and 9a participate in trafficking of the L2/vDNA complex to the TGN. (step 7ii) A portion of L1 protein is trafficked to the lysosome for degradation. (step 8) L2-containing vesicles derived from the Golgi interact with microtubules via exposed L2 domains facilitating Rab6a-dependent vDNA vesicle transport to the mitotic nucleus where the L2/vDNA complex gains access to PML bodies. (step 9) Thereafter, early viral transcripts are translated into early gene products, including E2, which tethers vDNA to mitotic chromosomes. (step 10) Localization to the mitotic chromosome grants the vDNA access to the cellular transcription and replication machinery and promotes the establishment and maintenance of vDNA in dividing cells. Image created with BioRender.

**FIGURE 2:**
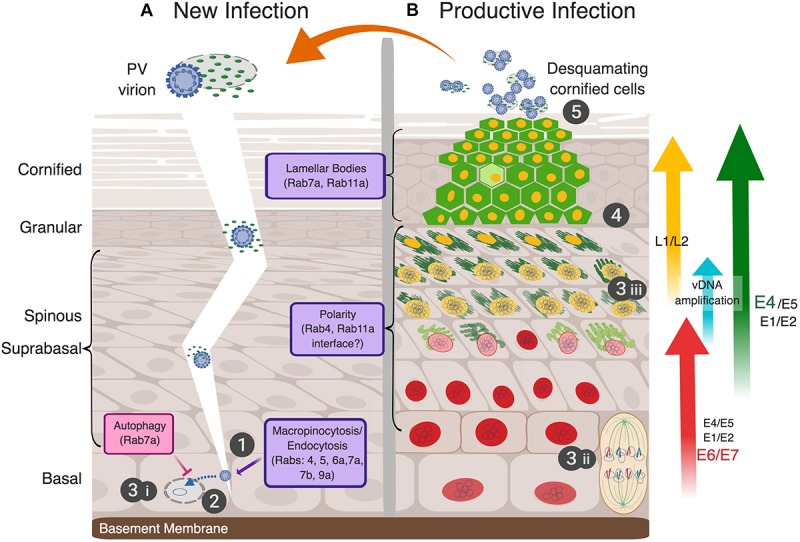
The full papillomavirus replicative-cycle requires stratifying and differentiating epithelium. The five canonical steps of virus infection as shown numbered (1-attachment, 2-entry, 3-genome replication, 4-assembly, 5-release). **(A)** Infectious virions (blue-studded, circular particles shown encapsidating the cell histone-bound double-stranded vDNA) in the context of desquamating cornified cells (DCCs) gain access to replication competent, mitotically active basal cells via an epithelial barrier breach to attach and initiate a new infection (step 1). Whether cellular or soluble virus factors, e.g., the viral E4 protein, play an active role in infection is unknown (depicted as a fragile DCC with viral E4 shown as small green ovals). As discussed in the text, PVs use endocytic vesicles for virion uptake to transport the viral genome to the nucleus (step 2), and thus, interface with Rab proteins directly and indirectly (noted in the lowest purple box). The first phase of viral genome replication in the nucleus is thereafter initiated (step 3i). **(B)** A simplified view of viral gene expression during a persistent infection, which is tightly linked to epithelial differentiation, is illustrated and explained more fully in the text. The epithelial layers are morphologically characterized as basal, spinous, granular, and cornified cells, above the basement membrane (left of panel A). The viral genome (shown as small, intranuclear blue circles) is replicated in three separate stages (steps 3i–iii): (3i) *establishment*, where the incoming viral genome is replicated to 10–50 copies per nucleus; (3ii) *maintenance*, wherein viral genomes are replicated with cellular DNA and partitioned into daughter cells mediated by E2 linkage to mitotic chromosomes; and (3iii) *amplification* in the upper suprabasal cell layers in preparation for the assembly of progeny virions. These three phases of vDNA replication, as well as early and late viral gene expression, are separated temporally and spatially in the productively infected epithelium (reviewed in Ozbun and Meyers, 1999; Graham, 2010). Expression of viral proteins denoted as E1, E2, E5, E6, and E7 are modulated during infection (Doorbar et al., 2012; Graham, 2017). Potential sites of viral interface with Rab proteins with epidermal functions in the suprabasal and granular layers are indicated with purple boxes. Red nuclei represent infected cells expressing the E6 and E7 proteins, cytoplasmic green represents E4 protein amyloid fibrils, with increasing green darkness representing increased expression levels. E4 plays an essential role in aiding virus release by disrupting cytokeratins to compromise the integrity of DCCs. Yellow nuclei demark the expression of capsid proteins L1 and L2, which encapsidate the vDNA into newly assembled virions (step 4). Infectious progeny virions are released from the apical layer of the epithelium associated with fragile DCCs to initiate a new round of infection (step 5). Image created with BioRender.

Nearly all mammalian DNA viruses, including PVs, must deliver their genomes to the host cell nucleus to begin their replicative cycles and have evolved to usurp Rab-GTPase functions and diverse intracellular endocytic trafficking routes to do so (Figure 1; Spearman, 2018). However, to initiate a productive infection that can lead to the release of progeny virions (i.e., the complete replicative cycle), the PVs must also traverse the epithelial barrier to gain entrance into their susceptible target host cells, the mitotically active, basal squamous cells (Figure 2A step 1). Wounding or micro-abrasion of the epithelium has long been known to be important in facilitating efficient PV infections *in vivo* (Shope and Hurst, 1933; Rous and Beard, 1934; Reuter et al., 2001). Yet, besides providing the incoming virions access to the basal cells, whether additional aspects of the wound response play roles in early infection has not been methodically assessed.

In general, a complete viral replicative cycle is divided into five discrete steps that include (1) virion attachment to susceptible cells, (2) cellular uptake or virion penetration into the cytoplasm, including release and trafficking of the genome to the replication site, (3) viral genome replication, (4) progeny virion assembly, and (5) release of infectious particles. However, as illustrated in Figure 2, compared to most animal viruses, the complete replicative cycles of PVs are quite complex and rely intimately on intracellular processes that are modulated as cells leave the basal epithelial layer and epithelial differentiation ensues (Doorbar et al., 2012). The first three steps of the HPV replicative cycle are typically investigated in subconfluent keratinocyte monolayer cultures to model basal cells (Figure 1). Rab-GTPases, as widely conserved membrane platforms responsible for the scheduling of vesicle formation for cargo delivery throughout the cell (Wandinger-Ness and Zerial, 2014), regulate HPV uptake and viral genome trafficking to the nucleus to initiate infection, as will be further described below. The circular, extrachromosomal (“episomal”) vDNA is replicated in three phases that are spatially and temporally separated in the epithelium. The first, establishment phase of newly delivered episomal vDNA replication results in a low copy number (≈10–50 copies per cell) in infected basal cells (Figure 2A step 3i). As a basal cell divides, maintenance of vDNA replication occurs with host genome duplication and leads to vDNA partitioning into daughter cells upon cell division (Figure 2B step 3ii). Dependent upon incompletely understood cell differentiation cues, vDNA amplification occurs in the middle-to-upper epithelial layers to facilitate viral progeny assembly (Figure 2B step 3iii). With continued epithelial tissue differentiation, the viral replicative cycle proceeds into the late phase where the differentiation-dependent, late promoter is activated. The late genes encode the self-assembling L1 major capsid protein, and the L2 minor capsid protein (Figure 2B step 4). HPV virions comprise 72 pentamers of L1 and 12–72 copies of L2, which is responsible for viral genome incorporation (Favre et al., 1975; Buck et al., 2008; Doorbar et al., 2012). HPV transmission involves the release of progeny virions associated with desquamating cornified cells (DCCs) from the apical epithelium of a productive lesion (Figure 2B step 5). This process may take a week or longer. Rab-GTPases play important roles in establishing cell polarity and have many functions in differentiating epithelium. Although the interface between HPV activities and Rab proteins has not been directly studied in this context, such investigations stand to enrich our understanding of how HPVs alter cell polarity and provide insight into Rab functions therein.

### The Challenges of Investigating Papillomavirus Uptake Into Host Keratinocytes *in vitro*

Difficulties in obtaining purified, high-titer infectious HPV stocks from epithelial tissues have impeded efforts to carry out microscopy and genetic assays to investigate early HPV infection events. The most medically relevant (i.e., the sexually transmitted) HPVs produce low numbers of infectious virions in human lesions (Ozbun and Kivitz, 2012).

The vast majority of reports providing insight into HPV entry mechanisms utilized viral particles isolated from monolayer cell cultures ectopically overexpressing HPV L1 and L2 capsid proteins. These viral particles include infectious pseudovirions (PsVs) that carry a “reporter” expression plasmid as a pseudogenome (Buck et al., 2004) and quasivirions (QVs) where viral capsids package wild-type or genetically modified HPV genomes (Pyeon et al., 2005). Unlike epithelial tissue-derived virions (described further below), HPV particle assembly in this system requires a maturation step to permit inter-L1 disulfide bond formation to condense and stabilize the capsid (Buck et al., 2005). This epithelial differentiation-independent system yields relatively pure virus particles of 10^10^–10^11^ viral genome equivalents per milliliters (vge/ml) that better facilitate attachment and entry studies, particularly those using microscopic localization and virus particle tracking.

Two additional methods used to obtain infectious HPV virions include experimental epithelial tissue models that are commonly used to study the complete virus replicative cycle. First, the grafting of human epithelial tissue explants beneath the renal capsule of immunocompromised mice has been used to propagate virion stocks from HPV11 and HPV16 (Kreider et al., 1987; Bonnez et al., 1998). Second, the culture of three-dimensional (3D)-organotypic epithelial (“raft”) tissues from keratinocytes has greatly benefited HPV research as the tissue stratification and differentiation achieved provides an environment permissive for the complete viral replicative cycle (McCance et al., 1988; Meyers et al., 1997). Over the past 25 years, an increasing number of publications describe HPV virion production in the raft tissue culture system from naturally infected cells (Dollard et al., 1992; Meyers et al., 1992; Ozbun, 2002a, b) and from human keratinocytes stably maintaining episomal HPV genomes (Meyers et al., 1997; McLaughlin-Drubin et al., 2003; Lee et al., 2004; Holmgren et al., 2005). The tissue differentiation environment supports a tissue-spanning redox gradient that facilitates HPV virion assembly and maturation in the uppermost cornified epithelial layers (Conway et al., 2009). Like human lesions, these experimental tissue models produce relatively low numbers of HPV virions, yielding stock titers of 10^7^–10^9^ vge/ml (Ozbun and Kivitz, 2012).

To date, no differences have been determined in comparing mature HPV virions from differentiation-independent models with those from differentiated epithelial tissue models. Yet, recognizing the rather unique transmission mode of HPV virions with unstable DCCs in the epithelium, certain caveats should be considered when studying laboratory produced virions. Extracting and isolating intracellular HPV virions from intact monolayer cells or from whole epithelial tissues may yield virus stocks that are fundamentally different from virions shed in the *milieu* of fragile DCCs. For example, HPV transmission *in vivo* might be augmented by the presence of viral non-structural proteins or cell factors, whereas highly purified virions might be stripped of these factors. Conversely, virion stocks processed from whole-cell or tissue extracts may contain factors that would not be present in DCCs. This commentary is intended for consideration and not meant to discount any of the findings described below.

## Rab-GTPases in HPV Infectious Entry

In this section, we will describe the interaction of HPV virions, QVs, or PsVs with cell uptake and trafficking machinery (Figure 1), where it is important to note that the only viral proteins thought to be present are the L1 and L2 capsid proteins. Rab-GTPases play critical roles in directing the uptake and trafficking of infecting viral particles and also have functions in regulating many of the cellular factors involved in this process.

### Primary Attachment

Human papillomavirus particles preferentially attach to heparan sulfonated proteoglycans (HSPGs) present in the extracellular matrix (ECM) and epithelial tissue basement membrane (Figure 1 step 1; Joyce et al., 1999; Culp et al., 2006a, b; Johnson et al., 2009). Laminin-332 (LN332, formerly laminin-5) is an ECM receptor for HSPGs primarily accounting for the attachment of HPV particles to the ECM and basement membrane. HPV particles also attach to HSPGs on the keratinocyte plasma membrane (Giroglou et al., 2001; Selinka et al., 2002; Shafti-Keramat et al., 2003; Culp et al., 2006a, b; Richards et al., 2013; Surviladze et al., 2013). Ionic interactions between negatively charged heparan sulfate (HS) polymers and positively charged L1 capsid motifs mediate their interaction (Richards et al., 2013; Guan et al., 2017; Figure 1 step 1). Syndecan-1 (Sdc-1), the main epithelial HSPG, is the primary glycosaminoglycan thought to be responsible for HPV capsid interactions with cells (Shafti-Keramat et al., 2003). HPV virion binding to HSPGs facilitates capsid processing by the host cell enzymes cyclophilin B (CyPB), kalkeriein 8 (KLK8), and the proprotein convertase, furin (Richards et al., 2006; Bienkowska-Haba et al., 2009; Cerqueira et al., 2015). HSPG-bound L1 proteins are cleaved by CyPB and/or KLK8 to expose a furin cleavage site in the N-terminal 12 amino acids of L2 (Richards et al., 2006). Furin cleavage of L2, which can occur at the plasma membrane, exposes the so-called RG-1 antibody epitope near L2’s N-terminus. However, the effects of preventing RG-1 epitope exposure are only manifest in the endosome where escape of the L2/vDNA complex (shown in Figure 1 step 6) is inhibited (Richards et al., 2006). After capsid “priming” at the cell surface, virions translocate to an incompletely defined entry receptor complex (Figure 1 step 2). Studies from our laboratory indicate that HPV infection is dependent upon matrix metalloproteinases (MMPs), ADAM sheddases, and heparinases to release HS-bound capsids from the ECM and plasma membrane to facilitate infection (Surviladze et al., 2012, 2015).

Rab-GTPases are involved in the regulation of Sdc-1 and MMP activities, which may influence the priming of HPV virions on the cell surface. Increased cleavage of the Sdc-1 ectodomain (termed “ectodomain shedding”) is regulated by Rab5, MMP9, and growth factors. Rab5-GDP directly interacts with the cytoplasmic tail of Sdc-1 at the plasma membrane. This interaction prevents Sdc-1 ectodomain shedding until Rab5-GDP exchange to Rab5-GTP via ras-related signaling mediated by growth factor receptors (GFRs), such as the epidermal GFR (EGFR), and heparinase (Hayashida et al., 2008). As will be discussed in more detail below, it is known that HPV PsVs activate EGFR signaling as a requirement for HPV infection (Schelhaas et al., 2012; Surviladze et al., 2012), and that Rab5 functions are critical for proper trafficking post-HPV entry (Table 1; Smith et al., 2008; Schelhaas et al., 2012). However, whether efficient HPV particle movement from Sdc-1 to the entry receptor complex is influenced by Rab5 activity or its Sdc-1 interaction has not been investigated. Rab40b plays a role in the intracellular trafficking of MMPs 2 and 9 to the plasma membrane (Jacob et al., 2013), yet whether Rab40b influences HPV infection has not been tested.

**TABLE 1:** Rab-GTPase and related GTPase Involvement in HPV infection^1^.

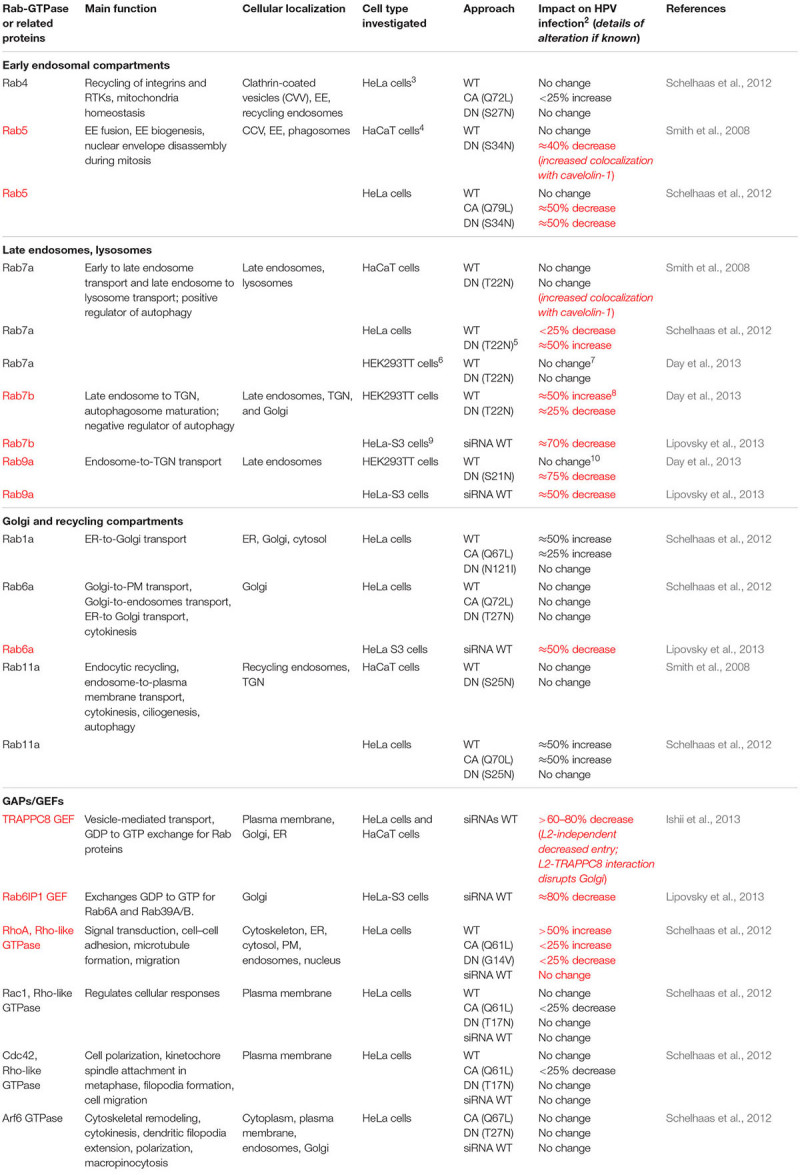

*^1^Positive effects are noted by red font. ^2^Relative to unaltered cells unless otherwise noted. ^3^Cervical carcinoma cells that express HPV18 E6 and E7 proteins. ^4^Spontaneously immortalized human keratinocytes. ^5^Mislabeled as S22N in the reference. ^6^Human embryonic kidney cells that express Adenovirus 5 E1A and E1b proteins and SV40 large T (tumor) antigen. ^7^Not compared to untransfected cells. ^8^Not compared to untransfected cells. ^9^A clonal derivative of HeLa cells. ^10^Not compared to untransfected cells*

Human papillomavirus exposure to keratinocytes activates EGFR signaling and leads to Src kinase-mediated phosphorylation of annexin A2 (AnxA2). This leads to the translocation of the AnxA2/S100A10 tetrameric complex (A2t) to the plasma membrane surface where HPV particles colocalize with EGFR and A2t (Dziduszko and Ozbun, 2013). Given the role of EGFR activity in Rab5-induced ectodomain shedding of Sdc1, it is plausible that this enhances the HPV virion translocation to the receptor complex. Thus, altered Rab functions may indirectly impact HPV capsid attachment and activation, thereby altering virion infectious potential.

### Entry Pathways

The current paradigm suggests that HPV uptake occurs via a receptor complex assembled by tetraspanin-enriched microdomains (TEMs) containing the tetraspanins CD151 and CD63, integrins, EGFR and A2t (Figure 1 step 2; Florin and Lang, 2018). HPV uptake occurs by an endocytic process similar to macropinocytosis, requiring actin polymerization, but ostensibly independent of clathrin, caveolin, flotillin, dynamin, and cholesterol (Schelhaas et al., 2012). Although the specifics of receptor-mediated HPV virion endocytosis have yet to be detailed, CD151, CD63, integrins, the A2t, the cytoskeletal associated adaptors obscurin-like protein (OBSCL1), and syntenin-1 are needed for efficient HPV particle uptake into cells (Figure 1 steps 2i,ii; Woodham et al., 2012; Dziduszko and Ozbun, 2013; Scheffer et al., 2013; Graessel et al., 2016; Taylor et al., 2018). Depletion of CD151 significantly reduced HPV16 internalization and largely prevented localization of HPV to EE (Scheffer et al., 2013). High-resolution microscopy revealed that HPV particles colocalize with CD151 at the plasma membrane. Only CD151-localized virions were able to enter cells and CD151 localized with HPV particles in intracellular vesicles (Spoden et al., 2008; Scheffer et al., 2013). Although not investigated in these reports, CD151 localizes with Rabs 4, 5, and 11 (Yang et al., 2002). Syntenin-1 and CD63 siRNA knockdowns reduced HPV infection by influencing endosomal trafficking and maturation in the early phases of viral uptake (Graessel et al., 2016). Immunoprecipitation of the syntenin-1/CD63 complex revealed interaction with HPV capsids; endosomal fractionation showed that HPV, syntenin-1, and Rab5 were colocalized, suggesting that syntenin-1 and Rab5 guide entering virions into developing EE structures (Graessel et al., 2016). TRAPPC8, a component of the guanine nucleotide-exchange factor (GEF) transport protein particle (TRAPP) complex that regulates small GTPases, was found to be required for HPV entry into cells (Ishii et al., 2013). A TRAPPC8 epitope was exposed on the plasma membrane and localized with HPV capsids independent of the presence of L2 protein in the virus particles. TRAPPC8 interacted with HPV L2, but only after capsid internalization. SiRNA-mediated knockdown of TRAPPC8 expression prevented HPV particle entry into cells and overall infection levels of both PsVs and virions from raft tissues (Ishii et al., 2013).

Additional plasma membrane-associated proteins have been shown to impact infectious HPV entry to some degree. These proteins include α6β4 integrin, the CD9 and CD81 tetraspanins, and EGFR (Figure 2 step 2; Evander et al., 1997; Abban and Meneses, 2010; Huang and Lambert, 2012; Schelhaas et al., 2012; Surviladze et al., 2012; Scheffer et al., 2013). EGFR signaling is clearly required for efficient HPV infection, but the specifics beyond activation of Src to recruit A2t and the need for PI3K and MEK signaling has yet to be determined (Schelhaas et al., 2012; Surviladze et al., 2012). Although AnxA2 is reported to facilitate endosome engulfment and the early endosome (EE) sorting of EGFR after ligand activation (Grewal and Enrich, 2009; Morel and Gruenberg, 2009), whether EGFR physically facilitates HPV entry is not currently clear. An AnxA2 antibody significantly blocked HPV entry into cells, whereas an antibody to the S100A10 subunit of A2t stranded a large number of HPV particles in the late endosome (LE)/lysosome; both antibodies inhibited infection (Dziduszko and Ozbun, 2013). These results are consistent with a role for A2t in endosome recruitment and association with a variety of interacting partners, including EGFR as noted above (Hubaishy et al., 1995; Bellagamba et al., 1997; Deora et al., 2004; Morel and Gruenberg, 2009; Zheng et al., 2011). Notably, uptake of each of the molecules identified as important for HPV infectious entry involve Rab5-positive endosomes in agreement with our observations and those of the Schelhaas Lab, that HPV infectious entry is dependent upon functional Rab5, detailed further below (Figure 1 step 3; Smith et al., 2007; Schelhaas et al., 2012).

### EE Entry and Acidification

Following conformational priming of the viral capsid at the cell surface and uptake, HPVs exploit EE acidification to promote capsid dissociation (Figure 1 steps 3,4; Smith et al., 2008). Virions enter CD63-positive EEs, potentially in an A2t-dependent manner, triggering vesicle-associated membrane protein (VAMP) associated protein (VAP)-dependent tubulation and endosomal acidification (Figure 1 steps 3,4) (Huotari and Helenius, 2011; Siddiqa et al., 2018b). VAP is important for actin nucleation and endosome-to-Golgi transport, and loss of VAP suppressed endosomal tubulation and reduced HPV trafficking to the *trans*-Golgi network (TGN) and infection (Siddiqa et al., 2018b). VAP mediates endoplasmic reticulum (ER) contacts with endosomes, the Golgi, and the plasma membrane. Recruitment of VAP to the ER-Golgi interface is regulated by the Rab3 GTPase activating protein 1/2 complex (Rab3GAP/2) (Hantan et al., 2014), implicating this Rab-GAP in HPV infection.

Rab5 is critical for endosomal biogenesis and recruits ATP-dependent ion channels that flood the endosome with protons to lower the pH. Rab5 is a key regulator of HPV infection. Rab5 knockdown resulted in decreased infection along with the loss of all endocytic vesicles beyond the EE and dominant negative (DN) Rab5 trapped virions in EE (Smith et al., 2008; Schelhaas et al., 2012). Rab5 activities in EEs are well understood and require many interacting factors. EE Rab5 recruits Rabaptin5, which is complexed with the Rab5 exchange factor, Rabex5. These complexes are thought to interact with Rab4-GTP orchestrating a GTPase cascade resulting in recruitment of endosomal sorting domains and machinery (D’Souza et al., 2014; Kälin et al., 2016). Additionally, Rab5 plays an important role in EE fusion events by generating phosphatidylinositol 3-phosphate and recruiting EE antigen-1 (EEA1) (Simonsen et al., 1998). Rab5 localizes with AnxA2 and EEA1 in EE (Morel and Gruenberg, 2009). Likewise, Rab5 regulates EGFR signaling, endocytosis, and trafficking at the plasma membrane and at the EE (Barbieri et al., 2004; Ceresa, 2006); however, it is unclear at which point(s) in the endocytic pathway Rab5 regulates EGFR movement. Rab5 family member Rab31 and its effector, EEA1, facilitate trafficking of ligand-bound EGFR from early to LEs. EGFR entry into Rab5 vesicles can then lead to either Rab11-, Rab22-, or Rab4-mediated endosomal recycling, or to Rab7a- and Rab31-facilitated entry into the LE (Figure 1 steps 5,6; Ceresa, 2006; Chua and Tang, 2014; Wang et al., 2017). Rab4 and Rab11 are dispensable for HPV infection, suggesting that successful HPV trafficking avoids recycling endosomes (Figure 1 step 5i; Smith et al., 2008; Schelhaas et al., 2012). Notably, EE-resident Rab21 and Rab22 are responsible for EE maturation, and have reported interaction with molecules important for HPV uptake. Rab21 supports integrin receptor internalization (Simonsen et al., 1998), whereas Rab22 has the highest sequence homology to Rab5, both localizing to EEs. Rab22 disturbance causes Golgi fragmentation and, despite its localization in the EE, plays a role in TGN transport (Kauppi et al., 2002). These latter Rabs have not been studied in the context of HPV infection.

### Rab Conversion and EE-to-LE Transfer

Human papillomaviruses have adapted to use the canonical endocytic process of EE maturation into LE to continue their journey to the nucleus (Figure 1 step 6). Rab conversion (or “switching”) generally occurs at the EE and is responsible for endosomal maturation to either LEs or recycling endosomes (Figure 1 steps 5,6i). Cargo sorting is generally mediated by Rab interactions, particularly Rab4, Rab5, Rab7a, and Rab11. Post-EE acidification Rab5 is replaced by Rab7a (Figure 1 step 5ii), which promotes the conversion from EE to LE (Kälin et al., 2016). The absence of Rab7a prevents the fusion of LE to lysosomes, ablating function, whereas Rab7a overexpression results in formation of large endocytic structures suggesting enhancement of cargo degradation (Bucci et al., 2000). Progression of EE to LE is generally defined by the formation of multivesicular bodies (MVBs) and eventual fusion with the lysosome (Figure 1 step 7i; Huotari and Helenius, 2011). The fact that HPV infection appears generally independent of Rab7a expression and is enhanced by a DN Rab7a (Table 1) is consistent with the need for viral escape from the LE prior to lysosome biogenesis (Smith et al., 2008; Schelhaas et al., 2012; Day et al., 2013). Rab conversion may be linked to the interaction of the ER with HPV-positive endosomes as EE–ER interaction contributes to ER-dependent scission of virion-positive vesicles that mature into LE/MVBs (Figure 1 steps 5,6; Siddiqa et al., 2018b).

During endosomal maturation, the L1 capsid partially dissociates from L2 complexed to vDNA (Figure 1 steps 5,6; DiGiuseppe et al., 2017). This is pH dependent and linked to A2t and γ-secretase activities (Bienkowska-Haba et al., 2012). L2/vDNA complexes are sorted into MVBs in a cyclophilin-dependent manner, avoiding lysosomal degradation, whereas a portion of L1 accumulates in the LAMP1-positive compartment for degradation (Smith et al., 2008; Bienkowska-Haba et al., 2012). The C-terminus of L2 contains a cationic cell penetrating peptide (CPP), that when activated by γ-secretase, facilitates anchoring of the L2/vDNA complex into the vesicle wall (Figure 1 step 6; Inoue et al., 2018; Zhang et al., 2018). A highly hydrophobic, transmembrane domain in L2 stabilizes the tethering of the L2/vDNA complex to the vesicle.

### LE to TGN

Rab7b and Rab9a contribute to trafficking from the LE to the TGN, and siRNA-mediated knockdown or expression of DN versions of both inhibit HPV infection (Day et al., 2013; Lipovsky et al., 2013). The HPV L2 CPP permits the extrusion of a small cytosolic domain of L2 that interacts with cytosolic retromer for trafficking to TGN, thus avoiding the lysosome (Figure 1 step 7ii; Inoue et al., 2018; Zhang et al., 2018). MVB interaction of HPV L2/vDNA with retrograde chaperones appears to be facilitated through VAP-mediated endosomal tubulation and ER contact, in addition to Rab6a, Rab7b, and Rab9a sorting (Lipovsky et al., 2013; Siddiqa et al., 2018b). Before delivery of viral cargo to the TGN, L2 binds sorting nexin (SNX) 27, retromer, and SNX17, a component of the retriever complex (Bergant Marušiè et al., 2012; Pim et al., 2015). Retromer-L2 interaction drives complexed vDNA to the TGN, which appears dependent on Rab7a-mediated recruitment of VPS35, a major retromer component (Seaman et al., 2009). Retromer assembly and localization to the MVB results in L2’s interaction with SNX27 and VPS26 forming a link between the intracellular chaperone and the L2/vDNA complex (Siddiqa et al., 2018b). Specifically, L2 interacts with retromer through SNX27 and retriever via SNX17 (Popa et al., 2015; Siddiqa et al., 2018b).

The L2/vDNA complex seems to enter the TGN by at least two routes, one that is Rab7b dependent and one that is Rab9a dependent. Rab7b resides prominently in the TGN and Golgi apparatus, functioning to transport vesicles toward the Golgi (Progida et al., 2012). SiRNA knockdown and DN Rab7b substantially impeded HPV infection (Smith et al., 2008; Schelhaas et al., 2012; Day et al., 2013; Lipovsky et al., 2013). As noted above, inhibiting Rab9a also significantly decreased HPV infection, highlighting the importance of LE-to-TGN trafficking (Figure 1 step 8; Day et al., 2013). With essential activity in endosomal recycling, Rab9a activities in HPV infection also may be linked to its role in furin transport to the TGN for secretion, reducing virion activation at the cell surface. Yet, inhibiting furin’s processing of L2 increases L2/vDNA localization to pre-TGN endosomal compartments, reducing infection (Richards et al., 2006). Abrogating Rab9a function may additionally reduce the proper sorting of virion-positive LEs as they traffic toward the TGN. Additionally, Rab6a and the GEF Rab6IP1 are involved in LE-to-TGN sorting, whereas their expression is important for HPV infection, how they participate has not been determined (Lipovsky et al., 2013).

### Activation of Autophagy Inhibits HPV Infection

Alternative to HPV transport to the TGN, the activation of macroautophagy, an autophagy variant, impairs HPV infection (Griffin et al., 2013; Surviladze et al., 2013). Autophagy, an evolutionarily conserved process, functions to degrade damaged organelles or proteins in the cytoplasm and serves as an intrinsic cellular defense to facilitate the capture and clearance of invading pathogens. A recent review details how HPVs subvert and manipulate keratinocyte autophagy during infection and cancer progression (Mattoscio et al., 2018). We and the Pyeon Lab showed that HPV infectivity is dramatically enhanced by biochemical inhibition or knockdown of essential autophagy genes (Griffin et al., 2013; Surviladze et al., 2013). We showed that HPV virion-activated EGFR signaling suppresses the autophagic response through the PI3K/Akt/mTOR pathway (Surviladze et al., 2013). In HeLa cells (which express HPV E6 and E7 proteins) electron microscopy analysis of autophagosomes upon HPV internalization demonstrated HPV particle-containing autophagosomes; biochemical inhibition of autophagy enhanced HPV infectivity (Ishii, 2013). These findings support autophagy’s involvement in intrinsic host cell defense against HPV infection. Several Rab-GTPases (Rab1, Rab5, Rab4, Rab7a, Rab8, Rab9a, Rab11, Rab24, Rab32, Rab33) are implicated in the regulation of autophagy at various stages. Of these, Rab7a’s role in autophagy is the best characterized. Rab7a is recruited to autophagosomes where it regulates the fusion with lysosomes (reviewed in Hyttinen et al., 2013). Of three studies employing Rab7a DN mutants, only one showed a moderate increase in HPV infection and found that overexpressing wild-type Rab7a slightly enhanced infection (Smith et al., 2008; Day et al., 2013; Lipovsky et al., 2013). It may be worth revising the roles of Rabs and their functional partners in autophagy-mediated control of HPV infectious entry. We speculate that the levels and activities of Rab7a versus those of Rab7b may be crucial in determining whether HPV virions are targeted to the autophagosome or to the LE.

### Transport From the TGN to the Nucleus

To utilize the cellular transcription and DNA replication machinery, the HPV vDNA must exit the TGN and enter the nucleus. The L2/vDNA complex enters and resides in the *cis* and *cis*-medial Golgi until the onset of mitosis (Day et al., 2013). The TRAPPC8 GEF, in addition to impacting HPV capsid endocytosis, has altered functions via interacting with the viral L2 protein post entry. The intracellular interaction between L2 and TRAPPC8 causes Golgi dispersal and alters normal TRAPPC8 functions in ER-to-Golgi transport. This may be a means by which L2 then directs the vDNA trafficking to the nucleus from the TGN (Ishii et al., 2013). To date, this is the only clear example of HPV alteration of Rab-related functions.

The egress of L2/vDNA from the Golgi to the nucleus is initiated by cell cycle progression and nuclear envelope breakdown during mitosis (Pyeon et al., 2009; Aydin et al., 2014). The Sapp laboratory found that the L2/vDNA complex thereafter remains associated with residual L1 protein and is encased in a Golgi-derived vesicle (DiGiuseppe et al., 2016, 2017). The cytoplasmic, C-terminus of L2 interacts with the dynein motor protein complex, directing L2/vDNA-positive vesicles toward the condensed chromosomes (Figure 1 step 8). Translocation of L2/vDNA vesicles occurs through the microtubule organizing center (MTOC), which facilitates function of the mitotic spindle. The MTOC delivers L2/vDNA to mitotic spindles where L2 interacts with the condensed chromatin via a chromatin-binding domain (DiGiuseppe et al., 2017). Rab6a trafficking with dynein motors to the MTOC is involved in the assembly and release of some human cytomegalovirus (Spearman, 2018). Thus, it is tempting to speculate that Rab6a’s important role in HPV infections involves a similar role in transporting L2/vDNA vesicles to the MTOC (Figure 1 step 8; Lipovsky et al., 2013). Additional Rab-GTPases are involved in cytoplasmic vesicle localization to mitotic spindles and in mitotic progression. Rab5 and Rab11 are active in vesicular trafficking, and Rab7a, Rab8a, Rab22, Rab24, Rab25, Rab35, and Rab37 appear involved in mitotic progression (Das et al., 2014). Thus, it is reasonable to think that other Rab proteins might have a part in the movement of L2/vDNA from the TGN to the nucleus.

### Intranuclear Activities

The intranuclear L2/vDNA complex is directed to promyelocytic leukemia (PML)- and nuclear domain 10 (ND10)-containing bodies (Day et al., 2004; DiGiuseppe et al., 2017). PMLs and ND10s are indispensable for virus genome transcription and viral gene expression (Figure 1 step 9). Recent data suggest that PMLs can associate with EEs during mitosis. The linking of PMLs to EEs may be important and provide further insight HPV’s reliance on Rab5 activities in infection (Palibrk et al., 2014).

In the last steps of HPV infectious entry, vDNA localization to ND10/PML bodies is thought to facilitate initial genome transcription, followed by vDNA replication to establish a persistent infection (Figure 1 step 10; Day et al., 2004). In earlier studies of high-risk HPV infection, we showed that viral mRNAs was detected as early as 4 h post-exposure (Ozbun, 2002a, b). Following expression of the early (E) viral proteins, E1 and E2 mediate vDNA replication to a low copy number per cell, as discussed in the next section (Figure 2B). Thereafter, E2 tethers the vDNA to cellular chromatin enabling viral genome partitioning between daughter cells upon cell division (McBride, 2013). As detailed in Figure 2B, the replicative cycle is then dependent upon epithelial differentiation for gene expression and cellular functions that promote the assembly and release of progeny HPV virions.

## Rab Proteins, Differentiating Epithelium and the HPV Replicative Cycle

Figure 2B illustrates a productive lesion, similar to a low-grade cervical neoplasia caused by high-risk HPV16. We focus on what is understood about high-risk HPV infections, as these viruses and their resulting lesions are the best studied (Doorbar et al., 2012). Viral gene expression is tightly controlled in persistently infected cells and throughout the productive replicative cycle (Figure 2A; Ozbun and Meyers, 1999; Graham, 2017). The extreme early and late PV stages are separated both spatially and temporally in the epithelium and do not appreciably occur in the same cells (Ozbun and Meyers, 1997; Doorbar et al., 2012). Many Rab-GTPases are differentially expressed during epithelial differentiation (Table 2; Uhlen et al., 2015) and Rab activities differ substantially in 2D versus 3D epithelial cultures (Ioannou and McPherson, 2016). Viral alteration of Rab functions is typically more pronounced in the later replicative stages during virion assembly. As a nonenveloped virus devoid of known glycoproteins, HPV replication has limited interface with cell membranes and likely fewer requirements for direct Rab interactions and/or functional manipulation.

**TABLE 2:** Rab expression in human skin and cervical epithelium.

**Stratified epithelial layer**	**Rab protein predominant expression^1^**
Basal	Rab1a, Rab1b, Rab5a, Rab5c, Rab6a, Rab7a, Rab9a, Rab11a, Rab14, Rab17, Rab21, Rab22a, Rab28, Rab34, Rab29, Rab31, Rab43
Suprabasal	Rab1a, Rab1b, Rab5c, Rab6a, Rab7a, Rab14, Rab43
Cornified	Rab7a^2^, Rab11^3^, Rab43

*^1^Compiled from the Human Protein Atlas (https://www.proteinatlas.org). ^2^Raymond et al. (2008). ^3^Reynier et al. (2016).*

Human papillomaviruses’ requirement for tissue-based differentiation and the technical challenges associated with studying membrane function therein have hampered studies of Rab functions during later replication stages. Yet, there is emerging evidence that three of the HPV nonstructural proteins, E5, E6, and E7, each of which have roles in transformation, modulate aspects of endocytic transport pathways (reviewed by Siddiqa et al., 2018a). Below we highlight the known HPV-Rab interactions and will suggest potential roles for Rab-GTPases during viral replication.

### The Productive HPV Replicative Cycle

In the HPVs’ strict dependence on normal cell functions, viral gene expression disrupts epithelial polarity to enhance cell proliferation in suprabasal cells (Thomas et al., 2008; Banks et al., 2012). As Rab-GTPases regulate epithelial polarity (Ioannou and McPherson, 2016; Parker et al., 2018), there are ample opportunities for HPV proteins to alter Rab functions. The late viral stages alter the morphology and function of terminally differentiating keratinocytes (Doorbar, 2013) where Rab-GTPases function to maintain epithelial barrier homeostasis (Raymond et al., 2008; Reynier et al., 2016).

The multifunctional viral oncoproteins, E6 and E7, are expressed at relatively low levels and predominantly in the lower-to-middle epithelial layers. Therein, they maintain and augment cell cycle and viral genome replicative capacity and promote proliferation as suprabasal cells begin the differentiation program (Thomas et al., 1999; Flores et al., 2000; Moody and Laimins, 2010). The understanding of how E6 and E7 reprogram infected cells has been stymied by difficulties in detecting the proteins in infected cells and tissues. The abilities of oncogenic HPV E6 to degrade p53 and E7 to inactivate pRb, which are key to the transformation capacity of high-risk HPVs, permit downstream effects of these viral proteins to be assessed as surrogates (Doorbar, 2013).

Studies of E6 and E7 cellular localization and how they alter cellular pathways has generally been limited to their ectopic overexpression in monolayer cells. The E6 oncoprotein is localized primarily to the nucleus, but it can also be detected in the cytoplasm (Howie et al., 2009). In cervical cancer-derived HeLa cells, the majority of endogenously expressed E6 is found in the membrane fractions, with lower levels detected in the cytosolic and nuclear compartments (Guccione et al., 2002; Kranjec et al., 2016). Proteomic analyses suggest that E6 interacts with several different components of the endocytic sorting machinery, including the retromer components (VPS26, VPS29, and VPS35) and SNX27 (Rozenblatt-Rosen et al., 2012; Belotti et al., 2013). The mechanisms by which E6 alters cellular trafficking pathways have yet to be determined. HPV E7 shuttles between the cytoplasmic and nuclear compartments (Guccione et al., 2002; Dreier et al., 2011; Laurson and Raj, 2011), where it functionally interacts with nuclear and cytoplasmic factors therein (Roman and Münger, 2013; Songock et al., 2017). E7 is not reported to have roles in the regulation of endocytic pathways. Both E6 and E7 can modulate autophagy (reviewed in Mattoscio et al., 2018); however, they appear to do so indirectly through transcriptional mechanisms.

### Epithelial Differentiation and HPV-Induced Polarity Alterations

While E7 stimulates S-phase entry with E6 in the suprabasal layers, high-risk HPV E6 proteins interact with a number of PDZ-domain-containing cellular proteins involved in cell polarity (Thomas et al., 2008; Banks et al., 2012). High-risk E6 proteins, via a class I PDZ-binding motif at their carboxy termini, promote degradation of the core polarity regulators hScrib, Dlg1, MAGI-1, and others. Limited work done in the context of a replicating HPV genome showed that E6 protein stability is enhanced by interacting with hScrib (Nicolaides et al., 2011). Additionally, HPV genome replicative abilities are compromised when the PDZ-binding motif of E6 is disrupted both in monolayer cells and HPV-infected 3D-organotypic epithelial tissue cultures (Lee and Laimins, 2004; Nicolaides et al., 2011). HPV-infected 3D-organotypic cultures and infected biopsy tissues typically demonstrate a thickening of the basal-like epithelial layers and the presence of nuclei throughout the suprabasal layers (Figure 2B). However, this phenotype is lost in organotypic cultures maintaining viral genomes harboring E6 defective for the PDZ-binding domain (Lee and Laimins, 2004). This underscores the need for HPV infections to alter epithelial polarity to promote cell proliferation. WNT signaling and nuclear β-catenin accumulation are enhanced by HPV E7, which downregulates E-cadherin expression in adherens junctions (Laurson et al., 2010); E6 also contributes to increased nuclear β-catenin accrual dependent upon its ability to degrade PDZ-containing proteins (Banks et al., 2012; Bonilla-Delgado et al., 2012). These functions further serve to promote the epithelial-to-mesenchymal transition and cell proliferation (Banks et al., 2012). Rab4 and Rab11a are involved in E-cadherin trafficking (Ho et al., 2016) and their overexpression in cancers leads to WNT signaling and nuclear β-catenin accumulation (Hou et al., 2016; Yu et al., 2016). Thus, these Rab proteins may be altered by HPV-mediated disruption of epithelial polarity. HPV infection, specifically the E6 and E7 proteins, can also dissociate the Hippo pathway from cellular polarity control by promoting YAP stability and nuclear import (He et al., 2015; Strickland et al., 2018). Interestingly, Rab11a promotes YAP nuclear transport in lung carcinoma cells (Dong et al., 2017), alluding to a potential intersection between E6/E7-mediated YAP nuclear import and Rab11a to stimulate cell proliferation. Thus, by deregulating epithelial polarity in multiple ways, the E6 and E7 proteins foster both vDNA replication and expansion of the number of HPV-infected cells.

Concomitant with altered cell polarity in the middle to upper epithelial layers, increased expression of the HPV E1 and E2 replicative proteins facilitate viral genome amplification to >1000 copies per cell nucleus (Figure 2B step 3iii; Ozbun and Meyers, 1998). This is enhanced by expression of HPV E4 and E5 proteins (Fehrmann et al., 2003; Genther et al., 2003; DiMaio and Petti, 2013; Doorbar, 2013; Egawa et al., 2017). E5 is a small, highly hydrophobic transmembrane protein, which has a cytoplasmic C-terminal tail (Krawczyk et al., 2011). In overexpression studies, E5 localizes primarily to the ER, but it can also be found in the Golgi, in perinuclear regions, and on the plasma membrane (Conrad et al., 1993). E5’s cellular localization and other overexpression studies suggest that some of its activity may be related to the trafficking of cytoplasmic membrane proteins, including the EGFR and the keratinocyte GFR (KGFR) (Tomakidi et al., 2000; Suprynowicz et al., 2010; Belleudi et al., 2011; Purpura et al., 2013). Recently, E5 has been classified as a viroporin, a channel-forming viral membrane protein, able to modulate ion homeostasis and to play a critical role in many processes, including vesicle trafficking and supporting the late stages of viral replication (Fehrmann et al., 2003; Wetherill et al., 2012). Proteomic analyses suggest that E5 may interact with proteins involved in regulating vesicular trafficking, including the Rab-GTPases (Rab18, Rab32, Rab34), SXN family members (SNX4, SNX14, SNX19), coatomer subunits (COPA, COPB, COPE), and VAPs (VAPA and VAPB), among others (Rozenblatt-Rosen et al., 2012). Additionally, E5 inhibition of KGFR interferes with the transcriptional regulation of autophagy (Belleudi et al., 2015). Whether Rab functions are altered by E5 has not been addressed but seems probable.

In the upper-suprabasal epithelial layers of epithelial tissues and 3D-organotypic tissue models, EGFR and phopho-ERK1/2 signaling gradually diminish (Nanney et al., 1986; Groves et al., 1992), paralleling the pattern of HPV oncogene expression in early neoplasia (Doorbar, 2005). Yet, the E6/E7-mediated nuclear import of YAP results in increased expression of EGFR ligands. Additionally, E5, E6, and E7 can augment GFR signaling, which may serve to intensify proliferative signaling by EGFR/ERK in infected versus uninfected tissues (He et al., 2015; Strickland et al., 2018). As E6/E7 expression is enhanced by EGFR/ERK signaling (Hu et al., 1997; Akerman et al., 2001), this positive feedback may attenuate differentiation, and promote vDNA amplification in these suprabasal epithelial layers.

### Late Viral Functions

Increasing differentiation in the upper suprabasal layers drives strong activation of the late viral promoter and heightened expression of the non-structural E4 gene and the capsid genes, L1 and L2 (Brown et al., 1996; Ozbun and Meyers, 1997; Doorbar, 2013). Assembly of infectious progeny HPV virions occurs in the nucleus of the upper layers (Figure 2B step 4). Within the L1–L2 protein coat, the viral genome is condensed by cellular histones (Favre et al., 1977). In the upper epithelial layers, heightened expression of the cytosolic HPV E4 protein is accompanied by E4 processing and assembly into amyloid-like fibers (Figure 2B steps 3iii,4). This leads to cytokeratin network destabilization, enhancing virus egress (Doorbar, 2013). These late viral stages modify the morphology and function of terminally differentiating keratinocytes (Doorbar, 2013), where Rab7a and Rab11a have clear functions in epithelial barrier homeostasis (Raymond et al., 2008; Reynier et al., 2016). Although the potential for HPV-mediated disruption or modification of Rab activities is present, it has not been investigated to date.

Progeny virions do not appear to be fully released from the top epithelial layers of DCCs, which can transmit infection (Bryan and Brown, 2001; Figure 2B step 5). The cytosolic HPV E4 protein is abundant in these cells and may play a role in transmission and environmental stability of the shed virions (Bryan and Brown, 2000, 2001). In the context of HPV transmission via a virion- and E4-laden DCC, whether the E4 proteins, or other viral or cellular debris factors, play any active role(s) in HPV uptake into cells has yet to be addressed.

## Conclusion

Hijacking of common host trafficking pathways is a common theme for many intracellular pathogens (Spearman, 2018). A growing body of evidence suggests a complex network of interactions between viruses and Rab proteins, with recent work confirming the requirement of Rabs, GAPs, and GEFs during HPV infection and differentiation-dependent replication (Table 1). A clearer understanding of specific roles that Rab5, Rab6a, Rab7b, Rab9a, TRAPPC8, and Rab6bIPI have in HPV infectious entry is likely to increase our understanding of how HPV navigates the intracellular highways to gain access to the nucleus and begin vDNA replication. Further, many logical interfaces in the HPV replicative cycle for the contributions of additional, uninvestigated Rab proteins and their effectors. Expansion of HPV research into networks to include other common host processes like autophagy will continue to close gaps in our understanding of cell biology and how HPVs alter or adapt to these cellular processes to benefit their replicative cycles. Future investigation into Rab-GTPase functions and their roles in cellular trafficking pathways may provide ample opportunity to understand and abrogate viral infections using molecular approaches targeting these processes.

## Author Contributions

All authors listed have made a substantial, direct and intellectual contribution to the work, and approved it for publication.

## Conflict of Interest Statement

The authors declare that the research was conducted in the absence of any commercial or financial relationships that could be construed as a potential conflict of interest.
